# JOINT TRAJECTORIES OF MULTIMORBIDITY AND FRAILTY AMONG OLDER AMERICANS

**DOI:** 10.1093/geroni/igad104.1656

**Published:** 2023-12-21

## Abstract

Little is known about how health status considering both multimorbidity and frailty develops over time. This study aims to investigate joint trajectories of multimorbidity and frailty and examine their associated factors among older American adults.We used data from the National Health and Aging Trends Study (2011–2019). Fourteen chronic conditions were ascertained each year. Frailty was measured annually using the Fried frailty phenotype. Group-based trajectory models were fitted to identify joint trajectories of multimorbidity and frailty. Multinomial logistic regression model was adapted to examine the association of baseline characteristics with the trajectories. We identified six distinct joint trajectories of multimorbidity and frailty, including four concordant and two discordant trajectories. Compared with the Newly-developing condition and Constant non-frailty group, increasing age, low education, dual Medicare-Medicaid status, former or current smoker, mild or severe insomnia symptoms, and bothersome or activity-limiting pain were significantly associated with an increased likelihood of belonging to the other five groups with higher levels of multimorbidity or frailty (all P< 0.05). Multiple distinct joint trajectories of multimorbidity and frailty offer complementary evidence to the heterogeneous patterns in aging, as well as inform clinicians, policymakers, and researchers to develop targeted interventions and provide comprehensive care management in community settings.

